# Hyaluronic acid-based hydrogels: As an exosome delivery system in bone regeneration

**DOI:** 10.3389/fphar.2023.1131001

**Published:** 2023-03-17

**Authors:** Huiling Deng, Jiecong Wang, Ran An

**Affiliations:** Department of Plastic Surgery, Union Hospital, Tongji Medical College, Huazhong University of Science and Technology, Wuhan, China

**Keywords:** hyaluronic acid, hydrogel, extracellular vesicles, exosomes, bone regeneration

## Abstract

Exosomes are extracellular vesicles (EVs) containing various ingredients such as DNA, RNA, lipids and proteins, which play a significant role in intercellular communication. Numerous studies have demonstrated the important role of exosomes in bone regeneration through promoting the expression of osteogenic-related genes and proteins in mesenchymal stem cells. However, the low targeting ability and short circulating half-life of exosomes limited their clinical application. In order to solve those problems, different delivery systems and biological scaffolds have been developed. Hydrogel is a kind of absorbable biological scaffold composed of three-dimensional hydrophilic polymers. It not only has excellent biocompatibility and superior mechanical strength but can also provide a suitable nutrient environment for the growth of the endogenous cells. Thus, the combination between exosomes and hydrogels can improve the stability and maintain the biological activity of exosomes while achieving the sustained release of exosomes in the bone defect sites. As an important component of the extracellular matrix (ECM), hyaluronic acid (HA) plays a critical role in various physiological and pathological processes such as cell differentiation, proliferation, migration, inflammation, angiogenesis, tissue regeneration, wound healing and cancer. In recent years, hyaluronic acid-based hydrogels have been used as an exosome delivery system for bone regeneration and have displayed positive effects. This review mainly summarized the potential mechanism of HA and exosomes in promoting bone regeneration and the application prospects and challenges of hyaluronic acid-based hydrogels as exosome delivery devices in bone regeneration.

## 1 Introduction

Bone regeneration is a complex, multi-stage physiological process involving a variety of cells, cytokines, chemokines, growth factors, and intracellular and extracellular signaling pathways (Dimitriou et al., 2011; Majidinia et al., 2018; Salhotra et al., 2020). Fracture healing is the most common form of bone regeneration in clinical settings. It consists of two main mechanisms: direct and indirect remodeling, with the latter predominating (Ferguson et al., 1999; Dimitriou et al., 2011; St-Arnaud and Naja, 2011). Direct remodeling usually requires anatomical reduction and strict stability. Once these conditions are met, the lamellar bone and the Haversian systems are able to regenerate with fewer calluses (Marsell and Einhorn, 2011). Indirect remodeling, on the other hand, is not required to meet the conditions of anatomical reduction and strict stability. It is mainly formed calluses through both intramembranous and endochondral ossification (Marsell and Einhorn, 2011; St-Arnaud and Naja, 2011). Because of the limited ability of bones to self-heal after injury, it is urgent for clinicians to repair large bone defects caused by various reasons such as fractures, traumatic injuries, tumor removal, and infection, and restore their function (Bai et al., 2018; Wang et al., 2019; Yu et al., 2020). It has been demonstrated that once the length of the bone defect exceeds 2 to 2.5 times the diameter of the damaged bone, the self-healing ability of bone tissue alone is not enough (Wiese and Pape, 2010). Hence, some additional clinical treatments are needed to keep the bone defect site stable and create a suitable microenvironment for bone regeneration, thus achieving better bone regeneration and functional reconstruction of the defect site (Elliott et al., 2016; Zhang et al., 2022). Currently, autografts and allografts remain the main methods for repairing bone defects (Dimitriou et al., 2011; Chen and Lv, 2018). Nevertheless, the limited graft supply, donor area complications, immune rejection, and spread of infectious diseases associated with bone grafting have prompted the search for new bone substitutes to achieve the regeneration and functional reconstruction of bone tissue (Polo-Corrales et al., 2014; Amirazad et al., 2022).

Extracellular vesicles (EVs), including exosomes, endosomes, microparticles, apoptotic bodies and other different subtypes, are nanoscale biological vesicles released by different cells (Lotvall et al., 2014; Teng and Fussenegger, 2020). Exosomes are EVs with an average diameter of 100 nm, which contain different components such as nucleic acids, proteins, lipids, and metabolites, depending on the various sources of cells (Kalluri and LeBleu, 2020). Recently, exosomes derived from different kinds of cells have shown enormous potential in bone regeneration and repair as a key element of cell-free therapy (Zhai et al., 2020). This cell-free therapy strategy based on exosomes not only avoids the immune risks relevant to cell therapy and improves the low homing efficiency of transplanted cells, but it also can achieve the repair and regeneration of the defective bone tissue by regulating the inflammatory response and promoting angiogenesis and osteogenesis (Eggenhofer et al., 2014; Sun et al., 2022; Zhang et al., 2022). And since exosomes do not have the ability to self-replicate, the risk of iatrogenic tumor formation is also greatly reduced (Sun et al., 2022). Additionally, exosomes have high stability, can better maintain biological activity, and also have an intrinsic homing effect that can target organs (Lou et al., 2017; Yang et al., 2020). Unfortunately, exosomes have a short half-life in the body and are quickly cleared through body fluids without encapsulation (Riau et al., 2019). Therefore, it is very necessary to find a delivery vehicle that can achieve sustained release of exosomes at the site of injury while maintaining the bioactivity of exosomes.

**FIGURE 1:**
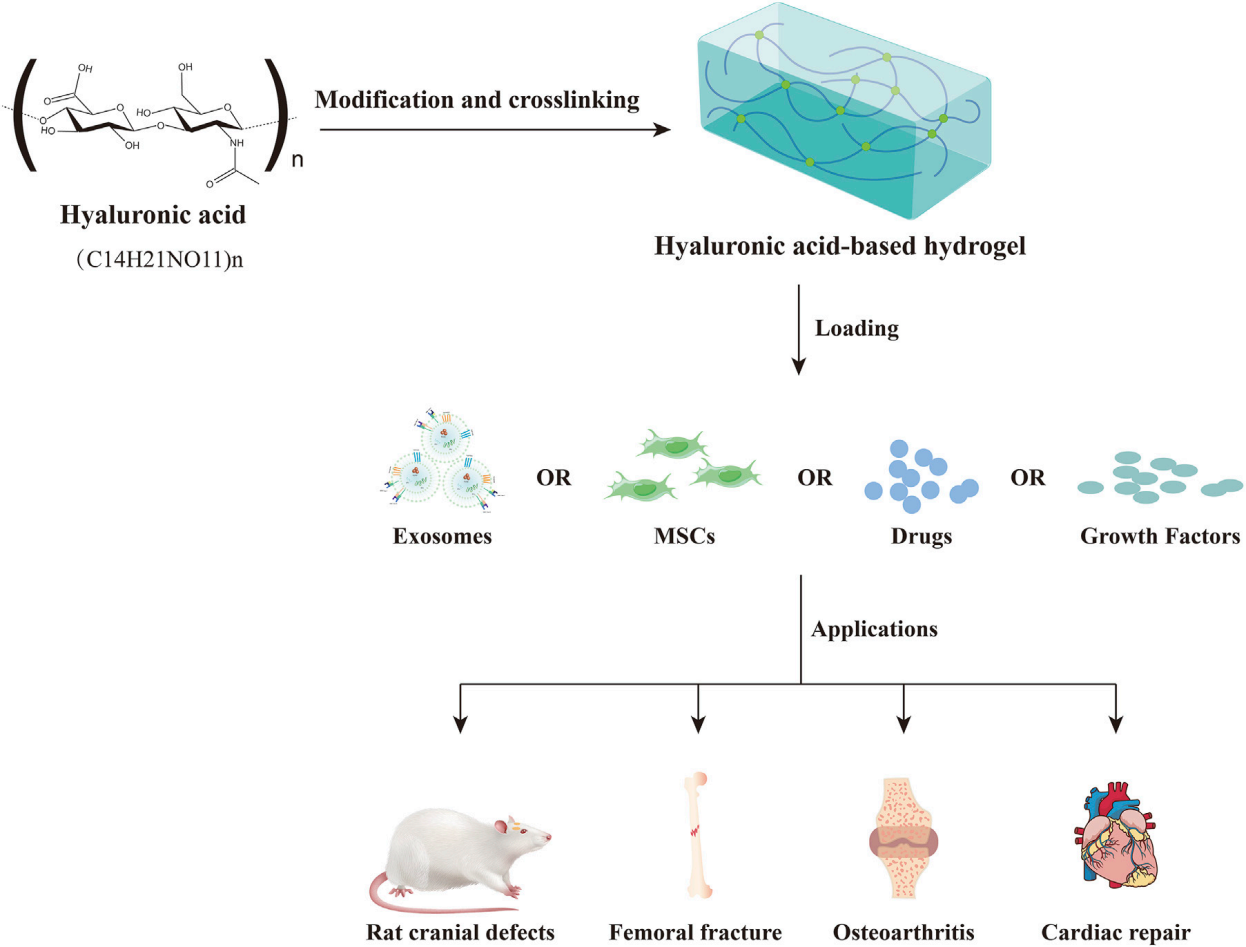
The applications of hyaluronic acid-based hydrogel as delivery vehicles in biomedical.

In recent years, hyaluronic acid-based hydrogels have received extensive attention for the delivery of exosomes from different cell sources (Xin et al., 2022). Hyaluronic acid (HA) is a naturally occurring unbranched glycosaminoglycan (GAG) that is composed of repeating units of the disaccharide β-1,4-D-glucuronic acid-β-1,3-N-acetyl-D-glucosamine (Burdick and Prestwich, 2011; Abatangelo et al., 2020). It is widely present in mammalian tissues and plays a critical role in cell differentiation, proliferation, migration, inflammation, angiogenesis, wound healing, cancer, diabetes and many other physiological and pathological processes (Passi and Vigetti, 2019; Marinho et al., 2021). Besides, hyaluronic acid-based hydrogels prepared by different physical or chemical methods as delivery vehicles for exosomes, mesenchymal stem cells (MSCs), drugs and growth factors, have been extensively studied in various disease models such as osteoarthritis, bone defects and cardiac repair (Figure 1) (Park et al., 2017; Jin Y. et al., 2020; Zhang et al., 2021b; Zou et al., 2021; Wang L. et al., 2022; Sang et al., 2022).

In this review, we described the synthesis and degradation of HA, the application of modified hyaluronic acid-based hydrogels in bone regeneration, the potential mechanism of hyaluronic acid in promoting bone regeneration, and the role of exosomes in bone regeneration. Finally, the applications, prospects and challenges of hyaluronic acid-based hydrogels as exosome delivery systems in bone regeneration were summarized.

## 2 Hyaluronic acid

### 2.1 Synthesis and degradation of HA

HA is a linear non-sulfated GAG formed by repeating disaccharides units (D-glucuronic acid and N-acetyl-D-glucosamine) linked alternately by β-1,3 and β-1,4 glycosidic bonds (Passi and Vigetti, 2019; Fang et al., 2021). Most cells in the body are capable of synthesizing HA (Marinho et al., 2021). Specially, the synthesis of HA differs from that of other types of GAGs, which are produced in the Golgi apparatus, whereas HA is produced by the hyaluronic acid synthases (HASes) in the inner leaflet of the cell membrane (Weigel et al., 1997; Passi and Vigetti, 2019; Abatangelo et al., 2020). The total content of HA in the human body is about 15 g, which is mainly in the skin and musculoskeletal tissues (Knopf-Marques et al., 2016). In mammals, HA is mainly synthesized by three types of HAS: HAS1, HAS2 and HAS3(Passi and Vigetti, 2019; Marinho et al., 2021). These three types of HAS use UDP-glucuronic acid and UDP-N-acetylglucosamine in the cytoplasm as substrates to catalyze repetitive sugar polymerization and extrude HA into the extracellular matrix (ECM) through the pore-like structures in the cell membrane (Vigetti et al., 2014; Kobayashi et al., 2020; Marinho et al., 2021). Due to the difference in the catalytic activities of these three types of enzymes, the molecular weight (MW) of the synthesized HA molecules ranges from 20,000 up to millions of Daltons (Vigetti et al., 2014; Trombino et al., 2019). For example, HAS1 and HAS2 can synthesize polymers with a high MW (larger than 2 × 10^6^ Da), while HAS3 synthesizes polymers with a low MW (1 × 10^5^ to 1 × 10^6^ Da) (Itano et al., 1999; Abatangelo et al., 2020). Moreover, the turnover of HA in the body is quick, the tissue half-lives range from several hours to a few days, and the daily turnover is about one-third of the total (Monslow et al., 2015; Trombino et al., 2019; Marinho et al., 2021). The HA in the blood is mainly degraded by the lymphatic system and liver, while HA in tissues is mainly degraded by hyaluronidases (HYALs), reactive oxygen species, superoxide dismutase, and nitric oxide produced by inflammation or damaged tissues (Collins and Birkinshaw, 2013; Garantziotis and Savani, 2019; Heldin et al., 2019; Abatangelo et al., 2020). In mammals, various HYALs such as HYAL 1-4, HYALP1 and PH-20 can degrade HA by catalyzing the hydrolysis of the β-1,4 glycosidic bonds between hexosamine and D-glucuronic acid residues (Stern and Jedrzejas, 2006; Stern et al., 2007; Knopf-Marques et al., 2016; Abatangelo et al., 2020). HYAL-1 and HYAL-2 are the main HYALs in mammals and widely exist in various somatic tissues (Kobayashi et al., 2020). PH-20 is the most active HYAL in mammals; it is mainly present on the surface of the sperm head and is essential for the fertilization of sperm (Weber et al., 2019; Kobayashi et al., 2020). These HYALs are capable of degrading HA into many small fragments. For example, HYAL-2 can break down HA into fragments of about 20 kDa, which are normally internalized into cells and subsequently further cleaved into smaller oligomeric HA by HYAL-1 in lysosomes (Tammi et al., 2001; Lee-Sayer et al., 2015; Abatangelo et al., 2020; Marinho et al., 2021). However, when the number of small fragments of HA in the ECM exceeds the clearance capacity of cells, these fragments can play an important role in biological processes such as angiogenesis and inflammatory responses (Bohaumilitzky et al., 2017; Passi and Vigetti, 2019). The balance between the synthesis and degradation of HA determines its MW and content, while the MW of HA determines its different biological functions (Abatangelo et al., 2020; Kobayashi et al., 2020; Marinho et al., 2021). For instance, the HA with high MW has angiogenesis-inhibiting, anti-inflammatory, immune-suppressive and cell proliferation-inhibiting effects, while the HA with low MW can induce extracellular matrix-associated cytokine activity, angiogenesis, tissue inflammation, and cell death (West and Kumar, 1989; McKee et al., 1996; Passi and Vigetti, 2019; Marinho et al., 2021; Ding et al., 2022).

### 2.2 Potential mechanism of HA in promoting bone regeneration

In order to exert its biological properties, HA needs to interact with receptors on the surface of the target cell to activate relevant signaling pathways within the cell. Presently, the interaction effects of a variety of receptors such as cluster of differentiation-44 (CD44), receptor for hyaluronic acid-mediated motility (RHAMM), cell migration inducing hyaluronidase 1 (CEMIP), hyaluronic acid-receptor for endocytosis (HARE), lymphatic vessel endothelial receptor-1 (LYVE-1), Toll-like receptors (TLR) and other receptors with HA have been widely researched (Garantziotis and Savani, 2019). CD44 and RHAMM are major HA receptors (Abatangelo et al., 2020). Previous research has established that the binding of HA and CD44 can affect various processes such as cell adhesion, bone metabolism, migration, inflammation, tumor growth and metastasis (Lesley et al., 1993; Ishida et al., 1997). Additionally, the study of Leng et al. (2019) suggested that CD44 and RHAMM have overlapping functions in regulating cell behavior and that HA binding to both receptors can regulate the motility and proliferation of myoblasts and connective tissue cells. Another study found that HA activates protein tyrosine kinases and ERK1, 2-MAP kinase cascades by interacting with CD44, RHAMM, and growth factors, thereby increasing the random movement ability of cells (Tolg et al., 2014; Knopf-Marques et al., 2016). Furthermore, it was shown that CEMIP, an HA-binding protein engaged in HA depolymerization, controls endochondral ossification through the metabolism of HA (Shimoda et al., 2017). Besides, high-MW HA can disrupt macrophage colony-stimulating factor signal transduction in osteoclast precursors and the TLR4, inhibit osteoclast differentiation, and thus prevent bone resorption *in vivo* (Chang et al., 2007).

Although the mechanism of interaction between HA and various receptors has been elucidated in many studies, the specific mechanism of HA in promoting osteogenesis still remains unclarified. Despite this, there are still studies constantly showing that HA is useful for the osteogenesis process. Previous studies have demonstrated that HA can limit osteoblast-mediated osteoclast genesis by interacting with CD44, thus playing an important role in bone metabolism and the communication between osteocytes and osteocytes or osteoclasts (Fujii et al., 2003; Kawano et al., 2011). It has also been shown that HA can bind to CD44 on the surface of human dental pulp stem cells (hDPSCs) and promote the expression of bone-related markers and osteogenic differentiation by inducing the YAP/TAZ pathway (La Noce et al., 2021). Moreover, the study by Huang et al. (2003) revealed that HA can increase the proliferation and differentiation of osteoprogenitor cells into the osteoblastic phenotype, as well as enhance the osteoinductive and osteogenic properties of bone graft materials and substitutes. This effect was dose-specific and molecular weight-specific; specifically, the HA with a high MW can increase the activity of alkaline phosphatase (ALP) and promote cell mineralization in a dose-dependent manner, while the HA with a low MW works well in increasing cell proliferation and the expression of osteocalcin mRNA (Huang et al., 2003). Recently, Lu et al. (2022) constructed an instantly fixable and self-adaptive hybrid cross-linked scaffold (HCLS) using dopamine-modified HA, micron hydroxyapatite, type I collagen and other materials to mimic the natural bone ECM. This scaffold not only has excellent deformation and mechanical matching ability but also provides an appropriate immune microenvironment to regulate the M2 phenotype polarization of macrophages. It has been demonstrated that the M2 macrophages play an active role in bone regeneration by secreting BMP-2, IL-10 and TGF-β to stimulate osteoblast differentiation and bone formation (Xiong et al., 2020; Li et al., 2021; Schlundt et al., 2021). Hence, when this scaffold is applied to cranial defect models in rabbits and beagle dogs, it can recruit endogenous stem cells *in situ*, rapidly initiate angiogenesis and osteogenesis, and accelerate osteogenic differentiation to promote cranial bone regeneration (Lu et al., 2022).

Additionally, HA also has an effect on angiogenesis, which is also essential for bone regeneration. Park et al. (2016) prepared a catechol-functionalized hyaluronic acid hydrogel loaded with human adipose-derived stem cells and applied it in the mouse hindlimb ischemia and critical-sized calvarial bone defects models, respectively. Their study illustrated that this hydrogel has excellent biocompatibility and tissue adhesiveness and can significantly promote angiogenesis and bone reconstruction at the defect site. Precious studies have shown that the Notch signaling pathway plays an important role in both bone development and remodeling (Zanotti and Canalis, 2016). It has also been demonstrated that Jagged-1 is the most highly expressed Notch ligand in fracture repair, and when it interacts with a membrane-bound receptor (Notch1-4) on the receiving cell, Notch signaling will be activated, thus promoting osteogenesis (Dishowitz et al., 2014). Consequently, Deng et al. (2021) conjugate the porous HA hydrogels with Jagged-1 ligand to enhance osteogenesis and bone regeneration by upregulating the Notch intracellular domain (NICD) and downstream msh homeobox 2 (MSX2, one of the Notch-signaling-related genes) to active Notch signaling. Furthermore, this functionalized Jagged-1 ligand can indirectly promote bone regeneration by regulating macrophage recruitment and promoting angiogenesis. Regrettably, although many previous studies have reported the ability of HA in promoting osteogenesis, the pathways and specific mechanisms involved in this process remain to be further investigated.

### 2.3 Application of modified hyaluronic acid-based hydrogels in bone regeneration

Although HA has great biocompatibility, biodegradability, and hydrophilicity, its mechanical properties are poorer than those of normal bone tissue, and the unmodified HA is susceptible to being degraded by HASes (Zhao et al., 2016; Fang et al., 2021; Pérez et al., 2021; Ding et al., 2022). Hence, in order to overcome these problems, researchers always modify HA and prepare it into hydrogel through various physical or chemical approaches to improve its mechanical properties, stability and half-life (Ahmadian et al., 2020; Marinho et al., 2021; Pérez et al., 2021). There are three types of sites in each disaccharide unit of HA that can be chemically modified, including carboxyl, hydroxyl and N-acetyl groups (Fang et al., 2021; Pérez et al., 2021). HA is mainly modified through its carboxyl groups, and the modification of its carboxyl groups using carbodiimide derivatives is one of the most widely used methods (Schanté et al., 2011; Pérez et al., 2021). Specifically, the carboxyl groups of HA can be amidated by 1- ethyl-3-[3-(dimethylamino)-propyl]-carbodiimide (EDC), 2-chloro-1-methylpyridinium iodide (CMPI), 2-chloro-dimethoxy-1,3,5-triazine (CDMT), 1,1′-carbonyldiimidazole, hexamethylenediamine (HMDA) and react with different crosslinking agents to generate hyaluronic acid-based hydrogels (Magnani et al., 2000; Park et al., 2002; Schanté et al., 2011; D'Este et al., 2012; Yan et al., 2012; Liang et al., 2015; Wang L. et al., 2018; Fang et al., 2021). Furthermore, it also can be esterified by alkyl halides, tosylate, diazomethane and epoxides, for tissue engineering and drug delivery (Burdick and Prestwich, 2011; Schanté et al., 2011; Collins and Birkinshaw, 2013). The hydroxyl groups of HA can form ether bonds under the modification of epoxides, divinyl sulfone, and ethylene sulfide, but they can also form ester bonds under the modification of octenyl succinic anhydride (OSA), activated compounds, and methacrylic anhydride (Schanté et al., 2011). The N-acetyl groups of HA can be deacetylated to form amino groups, which can react with the carboxyl group of HA to form an auto-crosslinked hydrogel or react with the active acid to form amide bonds (Schanté et al., 2011; Collins and Birkinshaw, 2013). For instance, the study of Zhang et al. (2020b) revealed that the HA modified by methacrylic anhydride can form lighted-cured hydrogels with good biocompatibility and mechanical properties in the presence of other crosslinking agents. In addition, Poldervaart et al. (2017) modified the naturally occurring extracellular matrix HA and prepared a photo-crosslinked methacrylated hyaluronic acid (MeHA) hydrogel with better mechanical strength and long-term stability using 3D bioprinting technology. In *in vitro* experiments, hydrogels with high concentrations of MeHA polymers can promote osteogenic differentiation of MSCs without additional osteogenic stimulation. Another study indicated that the combination of glycidyl methacrylate-modified hyaluronic acid-based hydrogels with appropriate osteoinductive molecules [such as morphogenetic protein 2 (BMP-2) and vascular endothelial-derived growth factor (VEGF)] can induce the formation of mineralized bone tissue and promote bone growth *in vivo* (Patterson et al., 2010). Moreover, the temporal progression of bone formation and remodeling can be regulated by changing the degradation rate of the hydrogel scaffolds (Patterson et al., 2010).

Currently, various crosslinking strategies for HA have been developed, such as click chemistry, Schiff base reactions, enzymatic crosslinking, disulfide crosslinking, radical polymerization crosslinking, and condensation reactions (Trombino et al., 2019; Ding et al., 2022). These hyaluronic acid-based hydrogels are widely used in tissue engineering, regenerative medicine, cell delivery, drug delivery and exosome delivery (Trombino et al., 2019; Bao et al., 2021; Flegeau et al., 2021; Hamilton et al., 2021; Yang et al., 2021; Cheng et al., 2022; Flegeau et al., 2022; Mi et al., 2022). Because of their high reactivity, selectivity, and yield, click chemistry strategies such as Diels–Alder reaction, Azide-Alkyn cycloaddition reaction, Thiol-ene reaction, Aldehyde-Hydrazide coupling and others have emerged as the most promising strategy for preparing hydrogels under mild conditions (Pérez et al., 2021). Since various modification and cross-linking strategies of HA have been described in detail in previous literature, this article will not introduce them further (Schanté et al., 2011; Collins and Birkinshaw, 2013; Khunmanee et al., 2017; Pérez et al., 2021; Di Mola et al., 2022). Table 1 lists various crosslinking strategies and applications of hyaluronic acid-based hydrogels.

**TABLE 1:** Crosslinking strategies and applications of hyaluronic acid-based hydrogels.

Methods	Features	Applications	References
Diels–Alder reaction	High biocompatibility; High efficiency; High electivity; Thermoreversibility; Mild reaction conditions; No toxic side products; Performed under physiological conditions; Without needing of toxic catalysts and initiators; Adjustable kinetics of crosslinking	Drug delivery; Cell delivery; Regenerative medicine; Tissue engineering; Mimicking extracellular matrix	Fisher et al. (2015), Wang et al. (2018a), Trombino et al. (2019), Cadamuro et al. (2020), Ilochonwu et al. (2022), Jo et al. (2022)
Azide-Alkyn cycloaddition reaction	High yield; High efficiency; High selectivity; Great bio-orthogonality; Fast reaction rate; High reactivity; Without needing of catalyst	Drug delivery; Regenerative medicine; As injection filling materials	Fu et al. (2017), Manzi et al. (2017), Trombino et al. (2019), Chen et al. (2020)
Thiol-ene reaction	High efficiency; High yield; High specificity; Fast reaction rate; Biocompatibility; Mild reaction conditions; Without needing of initiators; No toxic side products	Treatment of corneal inflammation; Promote diabetic wound healing; Cell encapsulation; Drug delivery; Regenerative medicine	Gwon et al. (2017), Trombino et al. (2019), Jin et al. (2020a), Zhang et al. (2020c), Park et al. (2022)
Aldehyde-Hydrazide coupling	High efficiency; Cytocompatibility; Without side products; Simple; Without cytotoxicity; Mild reaction conditions	Tissue engineering; Cell delivery; Cartilage tissue regeneration	Dahlmann et al. (2013), Chen et al. (2017), Trombino et al. (2019)
Schiff base reaction	Mild reaction conditions; High efficiency; Simple; Rapid reaction; Reversibility; Sensitive to changes in PH; Without crosslinking agents; Controllable reaction rates	Drug delivery; Cell delivery; Wound healing; Tissue regeneration; Bioprinting; Smart robots	Li et al. (2020), Lei et al. (2021), Ding et al. (2022)
Enzymatic crosslinking	Mild reaction conditions; High efficiency; Fast gelation rate; High specificity; High biocompatibility	Tissue engineering; As vitreous substitutes; Drug delivery	Trombino et al. (2019), Raia et al. (2020), Ziadlou et al. (2021), Ding et al. (2022), Wang et al. (2023)
Disulfide crosslinking	Safety; Easy to implement; Reversibility; *In-situ* gelation properties	As a scaffold for cell culture; Cell encapsulation; Gene delivery vectors; Drug delivery	Trombino et al. (2019), Wu et al. (2020)
Radical polymerization crosslinking	Excellent reaction kinetics; Easy to polymerize *in situ* in the presence of cells; Crosslinking reaction can be precisely controlled	Tissue engineering; Drug or biomolecule delivery	Bencherif et al. (2009), Das et al. (2018), Ding et al. (2022)
Condensation reaction	Without organic solvents and toxic substances required	Drug delivery; Wound dressings	Crescenzi et al. (2002), Trombino et al. (2019)

## 3 The role of exosomes in bone regeneration

**FIGURE 2:**
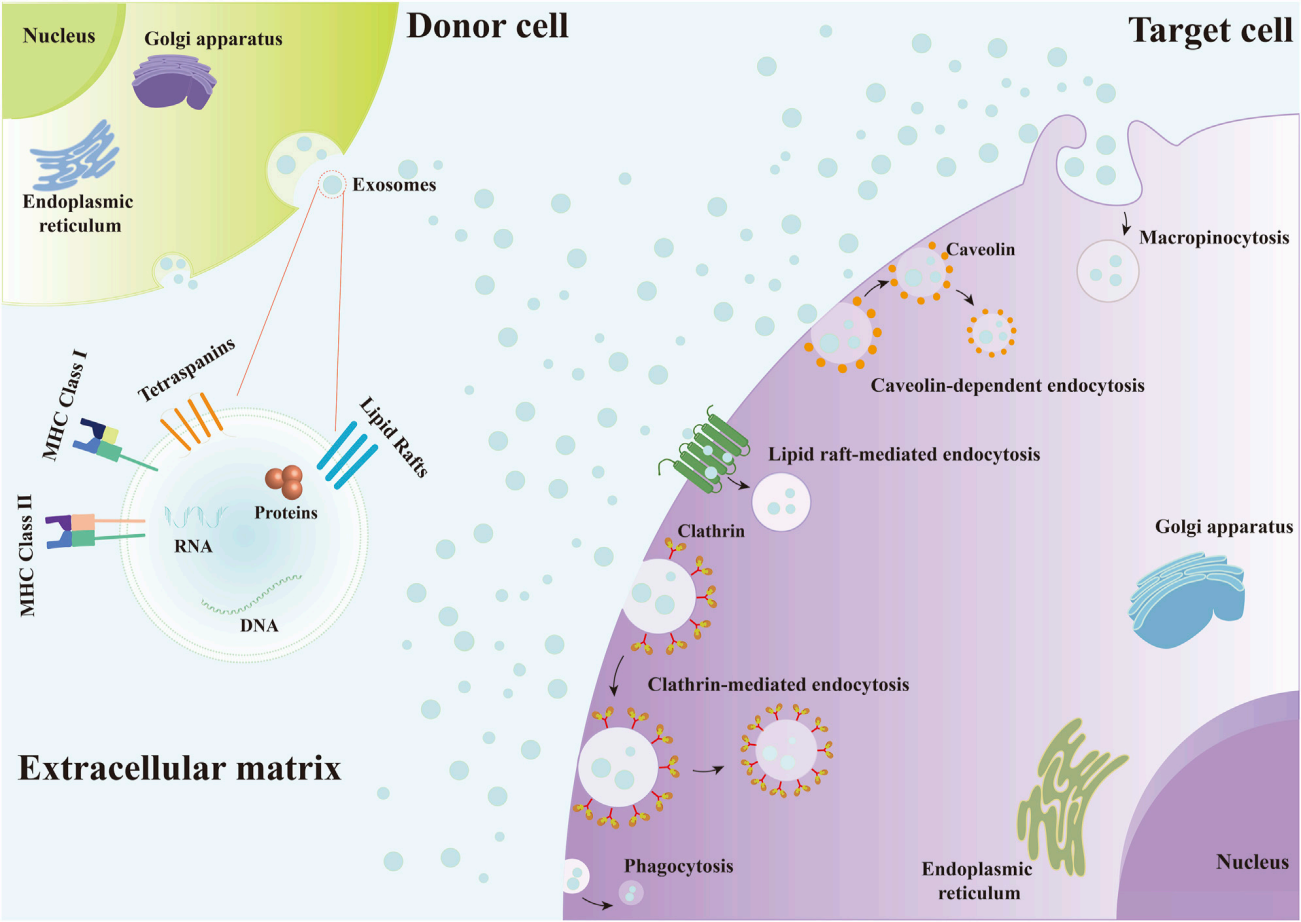
Exosomes enter target cells through various forms of endocytosis, including clathrin-mediated endocytosis, caveolin-dependent endocytosis, macropinocytosis and phagocytosis, and lipid raft-mediated endocytosis.

Exosomes are EVs that are released by various cell types and are widely found in the interstitial spaces and body fluids (Zhang et al., 2019b; Brigstock, 2021). Exosomes from various origins can influence gene expression and signaling pathways regulation in recipient cells by delivering biomolecules such as mRNA, miRNA, and proteins (Akbari et al., 2020; Kalluri and LeBleu, 2020; Hu et al., 2021; Sun et al., 2022). It has been demonstrated that tetraspanins, integrins, immunoglobulins, proteoglycans and lectins all play a role in the binding of EVs to recipient cells (Mulcahy et al., 2014; Hoshino et al., 2015; Mathieu et al., 2019). Exosomes can enter target cells through various forms of endocytosis, such as clathrin-mediated endocytosis, caveolin-dependent endocytosis, macropinocytosis and phagocytosis, and lipid raft-mediated endocytosis (Figure 2), and then release their contents (Mulcahy et al., 2014; Pegtel and Gould, 2019; Ginini et al., 2022; van Niel et al., 2022). And exosomes follow the endosome pathway after being internalized by the recipient cells, from early endosomes to late endosomes to MVBs (Gurung et al., 2021; Khan and Steeg, 2021). Eventually, some of them are degraded after fusion with lysosomes, while others escape the lysosomal pathway and transfer their contents into the cytoplasm of recipient cells, mediating the changes of recipient cells (Gurung et al., 2021; Khan and Steeg, 2021; van Niel et al., 2022). Moreover, exosomes can also fuse directly with the plasma membrane of recipient cells (Mulcahy et al., 2014; Mathieu et al., 2019; Teng and Fussenegger, 2020; Gurung et al., 2021; Kimiz-Gebologlu and Oncel, 2022). On the one hand, exosomes release their contents into the cytoplasm of recipient cells, and on the other hand, the direct interaction between exosomes and the receptors on the surface of recipient cells induces downstream signaling cascades (Gurung et al., 2021). Recently, exosomes have been widely used for bone regeneration due to their biocompatibility, biostability and low immunogenicity (Tan et al., 2020; Wang et al., 2020). In the bone defects microenvironment consisting of various cells, such as osteoblasts, osteoclasts, endothelial cells, immune cells, and MSCs, exosomes can participate in the regulation of angiogenesis, osteogenesis and inflammation by being phagocytosed by these target cells and interacting with them (Safari et al., 2021; Zhu et al., 2021; Zhang et al., 2022). For instance, Kang et al. (2022) designed an exosome-functionalized cell-free PLGA/Exo-Mg-GA MOF composite scaffold that simultaneously enhances angiogenesis, osteogenesis, and anti-inflammatory capacity. The human adipose stem cell-derived exosomes (hADSCs-Exos) released from this scaffold can enter the human bone marrow-derived mesenchymal stem cells (hBMSCs) and human umbilical vein endothelial cells (HUVECs) co-cultured with the scaffold through phagocytosis to promote osteogenic differentiation and accelerate bone reconstruction.

It was reported that exosomes secreted by MSCs derived from human induced pluripotent stem cells (hiPSCs) and the exosomes derived from human adipose stem cells, bone marrow mesenchymal stem cells (BMSCs), and other types of cells all have the potential to promote osteogenesis (Qi et al., 2016; Li et al., 2018; Chen et al., 2019; Zhang et al., 2020a). They can regulate the expression of osteogenic factors and osteogenesis-related proteins in the MSCs through the bioactive molecules they contain (Narayanan et al., 2016). Exosomes secreted by MSCs derived from hiPSCs (hiPSC-MSC-Exos), for example, not only affect *in vitro* proliferation and osteogenic differentiation of BMSCs by enhancing osteogenic gene and protein expression [such as Collagen type I (COL 1), Runt-related transcription factor 2 (RUNX-2) and ALP], but also stimulate bone regeneration of critical-sized calvarial defects in ovariectomized rats (Qi et al., 2016). And there are also studies demonstrating that exosomes secreted by MSCs derived from hiPSCs also can be internalized by BMSCs to enhance the osteoinductivity of beta-tricalcium phosphate (β-TCP) and promote the repair of calvarial bone defects in rats by regulating the PI3K/Akt signaling pathway (Zhang et al., 2016). Another study illustrated that optimized bone marrow mesenchymal stem cells-derived osteoinductive exosomes (BMSCs-OI-Exos) can enhance bone regeneration by targeting Acvr2b/Acvr1 *via* multiple miRNAs (such as let-7a-5p, let-7c-5p, miR-328a-5p, and miR-31a-5p) and activating Smad1/5/9 phosphorylation through Bmpr2/Acvr2b competitive receptors (Liu et al., 2021a). Moreover, there are also studies revealing that miR-5,106 in M2 macrophage-derived exosomes (M2D-Exos) can induce the osteogenic differentiation of BMSCs and promote femoral fracture healing in mice by targeting the salt-inducible kinases 2 and 3 (SIK2 and SIK3) (Xiong et al., 2020). And the study of Zhang et al. (2021a) indicated that bone marrow mesenchymal stem cells-derived exosomes (BMSCs-Exos) can also reduce the osteoporosis in rats by delivering miR-935 to osteoblasts and targeting signal transducer and activator of transcription 1 (STAT1), inhibiting its expression, and promoting the proliferation and differentiation of osteoblasts.

As is known to us, the lack of a vascular network at the bone defect will prevent the formation of new bone, and adequate blood supply is an important basis for bone generation (Wu et al., 2021; Zhang et al., 2022). Angiogenesis and osteogenesis are closely related, and only by improving both at the same time can the remodeling of new bone be better improved (Behera et al., 2021; Wu et al., 2021). For instance, Wang et al. (2022b) manifested that macrophages-derived exosomes (MФs-derived exosomes) can promote the migration of endothelial cells and enhance the expression of angiogenic-related genes, thus promoting angiogenesis, while increasing ALP activity and the expression of osteogenic-related genes in BMSCs. Besides, the study of Zhang et al. (2019a) showed that human umbilical cord mesenchymal stem cells-derived exosomes (huMSCs-Exos) can indirectly promote fracture healing by enhancing the angiogenic capacity of HUVECs through increasing the expression of VEGF and hypoxia inducible factor-1α (HIF-1α). And another study revealed that BMSCs preconditioned with a low dose of dimethyloxaloylglycine (DMOG), DMOG-MSC-Exos can activate the AKT/mTOR signaling pathway and enhance the bone regeneration at the site of calvarial defects in rats by promoting angiogenesis (Liang et al., 2019). There are also studies reporting that BMSCs-derived exosomes can activate the lnc-H19/Tie2-NO signaling pathway in the MSCs and endothelial cells (ECs) by regulating the expression of the angiogenic factor angiopoietin 1 (Angpt1), thus significantly promoting endothelial angiogenesis and osteogenesis (Behera et al., 2021). Moreover, Zhang et al. (2020a) also illustrated that BMSCs-derived exosomes can promote the osteogenesis and angiogenesis of a femoral non-union rat model through activating the BMP-2/Smad1/RUNX2, and HIF-1α/VEGF signaling pathways. Table 2 is a summary of the studies on the applications of exosomes in promoting osteogenesis and angiogenesis mentioned above.

**TABLE 2:** Summary of relevant studies on the application of exosomes in promoting osteogenesis and angiogenesis.

Exosomes	Recipient cell	Animal model	Scaffold material	miRNA/Pathway	Gene or protein expression	Function	References
hADSCs-Exos	HUVECs and hBMSCs	Rat cranial defects model	PLGA/Exo-Mg-GA MOF composite scaffold	Not mentioned	Up-regulating the expression of ALP, RUNX2 and OCN	Enhancing angiogenesis, osteogenesis, and anti-inflammatory	Kang et al. (2022)
hiPSC-MSC-Exos	BMSCs	Calvarial defects model of ovariectomized rats	hIPSC-MSC-Exos+β-TCP scaffold	Not mentioned	Up-regulating the expression of COL 1, RUNX2 and ALP	Stimulating bone regeneration and angiogenesis	Qi et al. (2016)
hiPSC-MSC-Exos	BMSCs	Rat calvarial defects model	hiPS-MSC-Exos/β-TCP combination scaffold	Enhancing the osteoinductivity of β-TCP and regulating the PI3K/Akt signaling pathway	Increasing the expression of ALP, OCN, RUNX2, COL 1	Enhancing bone regeneration	Zhang et al. (2016)
BMSC-OI-Exos	BMSCs	Rat cranial defect model	Hierarchical mesoporous bioactive glass (MBG) scaffold	Targeting Acvr2b/Acvr1 *via* let-7a-5p, let-7c-5p, miR-328a-5p, and miR-31a-5p	Up-regulating the expression of COL1, RUNX2, BSP, OCN, Bmpr1a and Bmpr2	Enhancing bone regeneration	Liu et al. (2021a)
				Activating Smad1/5/9 phosphorylation through Bmpr2/Acvr2b competitive receptors			
M2D-Exos	BMSCs	Mice femoral fracture model	Locally injected	Targeting SIK2 and SIK3 genes by delivering miR-5106	Increasing the expression of COL 1, ALP, OCN and RUNX2	Promoting femoral fracture healing	Xiong et al. (2020)
BMSCs-exo	Osteoblast cells (hFOB1.19)	Osteoporotic rat model	Intracavitary injections	Targeting STAT1, inhibiting its expression by delivery miR-935	Enhancing the expression of RUNX2 and ATF4; Increasing ALP activity	Promoting osteoblast proliferation and differentiation	Zhang et al. (2021a)
MФs-derived exosomes	BMSCs and ECs	Mice	Intracavitary injections	Targeting Akt1 by delivery mir-3473e	Up-regulating the expression of COL 1, RUNX2 and ALP activity	Promoting osteogenesis of BMSCs and angiogenesis of ECs	Wang et al. (2022b)
huMSCs-Exos	HUVECs	Rat femoral fracture model	HyStem-HP hydrogel (Catalog: GS315, Glycosan Biosystems, Salt Lake City, UT, United States of America)	Not mentioned	Increasing the expression of VEGF and HIF-1α	Enhancing osteogenic differentiation	Zhang et al. (2019a)
DMOG-MSCs-Exos	HUVECs	Rat calvarial defects model	Classical porous hydroxyapatite scaffolds	Activating AKT/mTOR pathway	Increasing the expression of p-AKT, mTOR, and p-mTOR	Enhancing the bone regeneration by promoting angiogenesis	Liang et al. (2019)
BMSCs-Exos	ECs	Osteoporotic Cystathionine β-synthase -heterozygous mice model	Locally injected	Activating the lnc-H19/Tie2-NO signaling pathway	Regulating the expression of Angpt1	Promoting endothelial angiogenesis and osteogenesis	Behera et al. (2021)
BMSCs-Exos	HUVECs	Femoral non-union rat model	Locally injected	BMP-2/Smad1/RUNX2 and the HIF-1α/VEGF signaling pathways	Increasing the expression of OCN, OPN, OGN, BMP-2, Smad1 and RUNX2	Promoting the osteogenesis and angiogenesis	Zhang et al. (2020a)

Additionally, exosomes can also serve as delivery vehicles for therapeutic drugs, targeting delivery small molecules or nucleic acid drugs to specific cells or tissues, thus increasing the local concentration of therapeutic drugs and reducing side effects (Liao et al., 2019; Chinnappan et al., 2020; Elsharkasy et al., 2020; Liang et al., 2020; Herrmann et al., 2021; Liang et al., 2021; Wang et al., 2021). For example, a new engineered exosome was constructed through encapsulating a plasmid carrying the VEGF gene into a chondrogenic progenitor cells-derived exosomes. Following by combining it with polycaprolactone 3D-printed porous bone scaffolds and applying it to rat radial defect model. This engineered exosome exhibited favorable osteogenesis and angiogenesis effects *in vivo* (Zha et al., 2021). Moreover, the role of exosomes as drug carriers has also been studied in other pathological conditions, such as malignant gliomas and colorectal cancer (Liang et al., 2020; Wang et al., 2021).

All in all, exosomes derived from various cells that have osteogenic potential not only can directly promote the bone regeneration by enhancing osteogenic or the expression of osteogenic-related genes but also can indirectly promote bone regeneration by promoting the angiogenesis in bone defect sites. In addition, exosomes can also be used to carry small molecules or nucleic acid drugs to the target cells, which can promote osteogenesis and angiogenesis.

## 4 Application of hyaluronic acid-based hydrogel-loaded exosomes in bone regeneration

**FIGURE 3:**
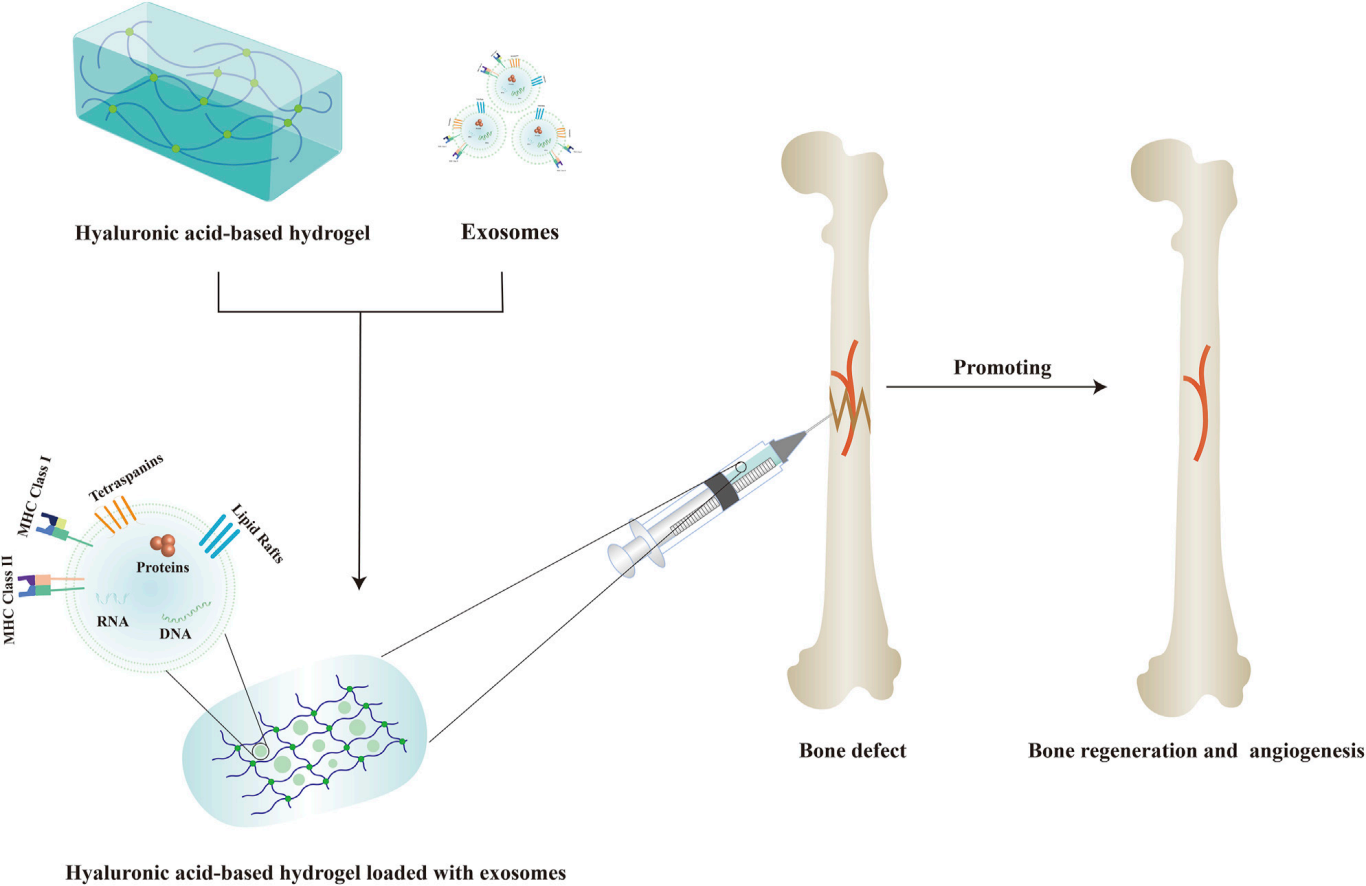
Schematic diagram of hyaluronic acid-based hydrogel loaded with exosomes for bone regeneration.

Hydrogel is a new type of biological scaffold composed of three-dimensional hydrophilic polymer chains with excellent biocompatibility, absorbability, and controlled mechanical properties that can mimic the natural ECM and provide a suitable growth environment for endogenous cells, and has been widely used in bone regeneration (Pishavar et al., 2021; Sun et al., 2022). Recently, various hyaluronic acid-based hydrogels have become a promising biomaterial and have been widely used in the biomedical field due to their biocompatibility, biodegradability, non-toxicity, high hydrophilicity, viscoelasticity, and other properties (Ahmadian et al., 2020; Ding et al., 2022). Although hyaluronic acid-based hydrogels have displayed promising effects in bone regeneration, it is not enough to implant a HA scaffold into the damaged area for tissue repair alone (Zhou et al., 2020; Deng et al., 2021; Arcos et al., 2022). Therefore, various cell-derived exosomes have been successfully introduced into hyaluronic acid-based hydrogels in order to better achieve bone regeneration at the defect areas. Figure 3 displayed the schematic diagram of hyaluronic acid-based hydrogel loaded with exosomes for bone regeneration.

It was reported that hyaluronic acid-based hydrogels loaded with exosomes can exert a variety of effects, such as promoting osteogenesis, angiogenesis and regulating inflammation response. For example, Yang et al. (2020) used an injectable hyaluronic acid-alginate (HA-ALG) hydrogel system with huMSCs-Exos and hydroxyapatite (HAP) encapsulated *in situ* to repair the cranial defects in rats. The *in vitro* experiments indicated that this HA-ALG hydrogel system with huMSCs-Exos and HAP encapsulated *in situ* can promote the proliferation, migration and osteogenic differentiation of the murine calvariae preosteoblast cell line (MC3T3-E1) by upregulating the expression of osteogenic-related genes and proteins such as ALP, osteocalcin (OCN) and Collagen type I alpha1(COL1A1). And in the rat cranial defect model, this HA-ALG hydrogel system encapsulating huMSCs-Exos and HAP can not only promote osteogenesis, but also effectively promote angiogenesis.

Additionally, vasculatures, as the major source of oxygen, nutrients, growth factors, immune cells, hormones and more, are indispensable in the repair and regeneration of various tissues (Liu et al., 2017; Chen et al., 2018; Mu et al., 2022). Zhang et al. (2021b) used hyaluronic acid-based hydrogels encapsulated with umbilical mesenchymal stem cells-derived exosomes (uMSCs-Exos) in combination with nanohydroxyapatite/poly-ε-caprolactone (nHP) scaffolds for the repair of cranial defects in rats. It has been shown that miR-21, the most abundant miRNA in uMSCs-Exos, can act as an intercellular messenger to promote angiogenesis by inhibiting the NOTCH1/DLL4 pathway. Furthermore, *in vitro* experiments have indicated that the uMSCs-Exos have no significant effect on the osteogenic differentiation of BMSCs, but they can indirectly promote bone formation by promoting angiogenesis and the proliferation and migration of the endothelial progenitor cells (EPCs).

Bone homeostasis is achieved by keeping the balance between osteoblasts and osteoclasts, which is strictly regulated by immune cells, endocrine system and osteocytes (Kong et al., 2019). And macrophages play a crucial role in bone homeostasis as a subpopulation of cells of the innate immune system (Kong et al., 2019; Li et al., 2019). When local stimulated by microenvironmental cytokines, macrophages can be divided into two phenotypes: M1 (secreting pro-inflammatory cytokines) and M2 (producing anti-inflammatory cytokines). And in the process of bone healing, only when the ratio of M1 and M2 macrophages reach a proper balance, can bone regeneration be better promoted (Li et al., 2019; Liu et al., 2021b). Previous studies have shown that M2 macrophages can upregulate ALP and increase bone mineralization, while M1 macrophages can inhibit osteoblasts and bone formation (Kong et al., 2019). Mi et al. (2022) designed a cocktail therapy to prepare an injectable hyaluronic acid-based hydrogel with anti-inflammatory, antibacterial, self-healing and tissue adhesion properties using aldehyde quaternary ammonium modified HA and hydrazine group modified HA. Subsequently, engineered ECs-derived exosomes (ECs-Exos ^miR-26a−5p^) and the inositol-requiring enzyme 1alpha (IRE-1α) inhibitor APY29 were loaded into the prepared HA hydrogels and injected *in situ* into the fracture site of a mouse model. In this way, the ECs-Exos^miR-26a−5p^ and APY29 loaded in the HA hydrogel can achieve sustained release and function at the injury site. Additionally, it can inhibit inflammatory cytokines and osteoclast differentiation and promote osteogenic differentiation and macrophage M2 polarization, showing an excellent effect in fracture repair (Mi et al., 2022). Table 3 is a summary of relevant studies on the application of hyaluronic acid-based hydrogels as exosomes delivery system in bone regeneration.

**TABLE 3:** Summary of relevant studies on the application of hyaluronic acid-based hydrogels as exosomes delivery system in bone regeneration.

Exosomes	Recipient cell	Animal model	Scaffold material	miRNA/Pathway	Gene or protein expression	Function	References
huMSCs-Exos	The murine calvariae preosteoblast cell line (MC3T3-E1)	Rat cranial defects model	HA-ALG hydrogel system with injectable HAP-embedded *in situ*	Not mentioned	Upregulating the expression of ALP, OCN and COL1A1	Promoting the proliferation, migration and osteogenic differentiation	Yang et al. (2020)
uMSCs-Exos	BMSCs and EPCs	Rat cranial defects model	Hyaluronic acid-based hydrogels encapsulated with uMSCs-Exos in combination with nHP scaffolds	miR-21/NOTCH1/DLL4 signaling axis	Upregulating the expression of proangiogenic genes, including VEGFA, CD31, and HIF-1α	Promoting angiogenesis and the proliferation and migration of EPCs	Zhang et al. (2021b)
engineered ECs-Exos ^miR-26a−5p^	BMSCs and bone marrow-derived macrophages (BMDM)	Mice femoral fracture model	Hyaluronic acid-based hydrogel preparation by aldehyde quaternary ammonium modified HA and hydrazine group modified HA	miR-26a-5p and APY29	Increasing the expression of COL1A1, ALP, OCN and RUNX2	Inhibiting inflammatory cytokines and osteoclast differentiation and promoting osteogenic differentiation and macrophage M2 polarization	Mi et al. (2022)

## 5 Conclusion and perspectives

With the development of new crosslinking strategies and the improvement of existing ones, more and more effective hyaluronic acid-based hydrogels for exosome delivery will emerge. By changing the concentration and molecular weight of HA polymer, hyaluronic acid-based hydrogels with different viscoelasticity and mechanical properties can be obtained to better meet the needs of different tissue repair (Passi and Vigetti, 2019; Fang et al., 2021). Additionally, the research on the bone regeneration microenvironment continues to deepen, and more bone regeneration strategies will appear in the future. For example, by adding different therapeutic components to the hydrogels, the hydrogels can have different effects on various processes such as inflammation, osteogenic differentiation, angiogenesis, and immune regulation in the bone regeneration process, thus promoting bone regeneration more efficiently. In this review, we summarized the use of hyaluronic acid-based hydrogels as exosome delivery systems for bone regeneration. Hyaluronic acid-based hydrogels have good biocompatibility, non-toxicity, controlled degradation, injectability, and adjusted mechanical properties. Besides, the hyaluronic acid-based hydrogels loaded with growth factors, exosomes or other therapeutic components can also achieve sustained release and action of exosomes and other therapeutic components at the site of the injury, which has good prospects for bone regeneration. However, natural HA cannot form hydrogel alone due to its high hydrophilicity and susceptibility to degradation by HASes. Therefore, it is often necessary to modify and crosslink natural HA to obtain hyaluronic acid-based hydrogels that can be used in tissue repair and regeneration. The development of different modification and crosslinking strategies has greatly improved the stability and mechanical properties of hyaluronic acid-based hydrogels, but there are still many challenges in their biomedical application and clinical translation. Firstly, the current modification or crosslinking strategies of HA may involve toxic catalysts or initiators or produce toxic by-products. Consequently, it is essential to develop new and better HA modification and crosslinking strategies. Secondly, the interactions and influence between the obtained hyaluronic acid-based hydrogels and the exosomes they loaded as well as the surrounding tissues need to be further explored. In addition, more studies are needed to clarify the role and mechanism of exosomes from different call sources in bone regeneration.

## Glossary

EV: extracellular vesicle
ECM: extracellular matrix
HA: hyaluronic acid
GAG: glycosaminoglycan
MSC: mesenchymal stem cell
HASes: hyaluronic acid synthases
MW: molecular weight
HYALs: hyaluronidases
CD44: cluster of differentiation-44
RHAMM: receptor for hyaluronic acid-mediated motility
CEMIP: cell migration inducing hyaluronidase 1
HARE: hyaluronic acid-receptor for endocytosis
LYVE-1:Lymphatic Vessel Endothelial Receptor-1
TLR: Toll-like receptors
hDPSCs: human dental pulp stem cells
ALP: alkaline phosphatase
HCLS: hybrid cross-linked scaffold
NCID: Notch intracellular domain
MSX2: msh homeobox 2
EDC: 1- ethyl-3-[3-(dimethylamino)-propyl]-carbodiimide
CMPI: 2-chloro-1-methylpyridinium iodide
CDMT: 2-chloro-dimethoxy-1,3,5-triazine
HMDA: hexamethylenediamine
OSA: octenyl succinic anhydride
MeHA: methacrylated hyaluronic acid
BMP-2: morphogenetic protein 2
VEGF: vascular endothelial-derived growth factor
PLGA: Poly (lactic acid-co-glycolic acid)
GA: gallic acid
MOF: metal-organic framework
hADSCs-Exos: human adipose stem cell-derived exosomes
hBMSCs: human bone marrow-derived mesenchymal stem cells
HUVECs: human umbilical vein endothelial cells
hiPSCs: human induced pluripotent stem cells
BMSCs: bone marrow mesenchymal stem cells
hiPSC-MSC-Exos: exosomes secreted by mesenchymal stem cells derived from human induced pluripotent stem cells
COL 1: Collagen type I
RUNX-2: Runt-related transcription factor 2
β-TCP: beta-tricalcium phosphate
BMSCs-OI-Exos: BMSCs-derived osteoinductive exosomes
Acvr2b: activin A receptor, type IIb
Acvr1: activin A receptor type 1
Bmpr2: bone morphogenetic protein receptor 2
M2D-Exos: M2 macrophage-derived exosomes
SIK2: salt-inducible kinases 2
SIK3: salt-inducible kinases 3
BMSCs-Exos: BMSCs-derived exosomes
STAT1: signal transducer and activator of transcription 1
**MФs-derived** exosomes: macrophages-derived exosomes
huMSCs-Exos: human umbilical cord mesenchymal stem cells-derived exosomes
HIF-1α: hypoxia inducible factor-1α
DMOG: dimethyloxaloylglycine
DMOG-MSC-Exos: BMSCs preconditioned with a low dose of DMOG
AKT: protein kinase B
mTOR: Mechanistic target of rapamycin
Tie2: Tyrosine-protein kinase receptor 2
NO: nitric oxide
lnc-H19: long non-coding RNA-H19
ECs: endothelial cells
Smad1: SMAD family member 1
Angp1: angiogenic factor angiopoietin 1
MBG: mesoporous bioactive glass
PI3K: Phosphoinositide 3-kinase
Smad5: SMAD family member 5
Smad9: SMAD family member 9
BSP: bone sialoprotein; Bmpr1a
ATF4: activating transcription factor 4
p-AKT: phosphorylated Akt
p-mTOR: phosphorylated-mTOR
OCN: osteocalcin
OGN: Osteoglycin
OPN: osteopontin
BBB: blood brain barrier
5-FU: 5-fluorouracil
HA-ALG: hyaluronic acid-alginate
HAP: hydroxyapatite
COL1A1: Collagen type I alpha1
uMSCs-Exos: umbilical mesenchymal stem cell-derived exosomes
nHP: nanohydroxyapatite/poly-ε-caprolactone
DLL4: Delta-like ligand 4
EPCs: endothelial progenitor cells
IRE-1α: inositol-requiring enzyme 1alpha
VEGFA: vascular endothelial growth factor A
